# Myocardial strain-curve deformation patterns after Fontan operation

**DOI:** 10.1038/s41598-023-39226-y

**Published:** 2023-07-24

**Authors:** Michal Schäfer, Max B. Mitchell, Benjamin S. Frank, Alex J. Barker, Matthew L. Stone, James Jaggers, Johannes C. von Alvensleben, Kendall S. Hunter, Richard M. Friesen, D. Dunbar Ivy, Roni Jacobsen, Michael V. Di Maria

**Affiliations:** 1 Division of Pediatric Cardiology, Children’s Hospital Colorado, Heart Institute, University of Colorado Denver, Anschutz Medical Campus, 13123 E 16th Ave, Aurora, CO USA; 2 Division of Cardiothoracic Surgery, Children’s Hospital Colorado, University of Colorado Denver, Anschutz Medical Campus, Aurora, CO USA; 3 Department of Radiology, University of Colorado Denver, Anschutz Medical Campus, Aurora, CO USA; 4 Department of Bioengineering, University of Colorado Denver, Anschutz Medical Campus, Aurora, CO USA

**Keywords:** Cardiology, Biomedical engineering

## Abstract

Myocardial deformation analysis by cardiac MRI (CMR) yielding global circumferential and longitudinal strain (GCS and GLS) is an increasingly utilized method to accurately quantify systolic function and predict clinical events in patients with Fontan circulation. The purpose of this study was to use principal component analysis (PCA) to investigate myocardial temporal deformation patterns derived from strain–time curves to learn about latent strain features beyond peak values. We conducted the study with specific attention to dominant single left or right ventricle (SLV and SRV) morphologies. Methods and Results: Patients remote from Fontan operation who underwent follow-up CMR were analyzed for standard volumetric and function hemodynamics including myocardial deformation parameters including GCS and GLS. We applied PCA to investigate in an unbiased fashion the strain–time curve morphology and to calculate patient specific shape scores. All variables were subjected to single variable Cox regression analysis to detect composite clinical outcome including death, heart transplant, protein losing enteropathy and plastic bronchitis. A total of 122 patients, (SLV = 67, SRV = 55) with a mean age of 12.7 years underwent comprehensive CMR analysis. The PCA revealed 3 primary modes of strain-curve variation regardless of single ventricle morphology and type of strain investigated. Principle components (PCs) described changes in (1) strain–time curve amplitude, (2) time-to-peak strain, and (3) post-systolic slope of the strain–time curve. Considering only SLV patients, GCS was only CMR variable predictive of clinical events (HR 1.46, *p* = 0.020). In the SRV group, significant CMR predictors of clinical events were derived indexed end-diastolic (HR 1.02, *p* = 0.023) and end-systolic (*HR* 1.03, *p* = 0.022) volumes, GCS (*HR* 1.91, *p* = 0.003) and its related first component score (HR 1.20, *p* = 0.005), GLS (HR 1.32, *p* = 0.029) and its third component score (HR 1.58, *p* = 0.017). CMR derived global strain measures are sensitive markers of clinical outcomes in patients with Fontan circulation, particularly in patients with the SRV morphology. Myocardial strain–time curve morphology specific to SLV and SRV patients inspired by unbiased PCA technique can further aid with predicting clinical outcomes.

## Introduction

Myocardial deformation analysis by cardiac MRI (CMR) derived strain is an increasingly used modality to assess the ventricular function in patients undergoing Fontan palliation^1,2^. Two -dimensional CMR feature tracking methods describing either global circumferential or longitudinal strain (GCS and GLS) benefit from excellent spatial resolution without acoustic window limitations^3^. This is a great advantage in patients with complex ventricular morphology undergoing surgical palliation resulting in myocardial scarring and dyssynchrony^4,5^. Most importantly, myocardial strain is a more sensitive marker of systolic function than ejection fraction (EF) which is confounded by ventricular thickness and volume^6^. As a result, strain-based measurements have been gaining popularity in studies attempting to detect subtle, subclinical changes in ventricular contractile function^2^. However, myocardial deformation temporal patterns in patients with univentricular morphology remote from of Fontan palliation have not yet been investigated.

The feature of strain–time curve with the best established clinical and physiologic relevance is the peak magnitude but other latent features of strain curves are largely unexplored. Global myocardial strain–time curve patterns have been recently qualitatively investigated in patients with pulmonary arterial hypertension with the goal to phenotype right ventricular diastolic function^7^. Myocardial segment specific strain–time curves are also used to describe classic-pattern dyssynchrony^8^, recently also applied in patients after Fontan operations^5^. The qualitative approach to categorize global ventricular strain–time patterns is ideal for patients with morphologically normal ventricle but is less ideal in patients with complex single ventricle anatomy. Our group has recently employed principal component analysis (PCA) as a method to study variations in physiologic signals such as flow-time curve patterns in Fontan circulation^9,10^. This approach provides an unbiased description of the most prevalent modes of standardized time-dependent signals, such as strain curves, which can later be associated with physiologic events occurring throughout the cardiac cycle. Given the increasing clinical utilization of global myocardial strain in clinical studies and its superiority as an index of systolic performance, computationally derived qualitative descriptors of strain–time curves in patients post Fontan operation might provide additional clinical and pathophysiologic insights into single ventricle function.

Consequently, our aim was to employ PCA to investigate myocardial deformation patterns derived from feature tracking CMR strain curves in patients late after Fontan operation, with specific attention to dominant single left or right ventricle (SLV and SRV) morphologies. Moreover, we sought to investigate the relationship between global strain indices and standard CMR ventricular parameters including EF and volumetric indices. Lastly, we studied whether are derived CMR derived hemodynamic parameters and strain measurements were predictive of clinical outcomes. We hypothesized that traditional strain-based indices as well as strain curve morphology will be associated with the single ventricular function and the overall clinical outcomes.

## Methods

This was a single center retrospective cohort study of patients who underwent single ventricle palliation, culminating in a Fontan operation, and were referred to the Single Ventricle Care Program, Multidisciplinary Clinic, at Children’s Hospital Colorado between December 2013 and September 2022. Per the institutional protocol, patients underwent comprehensive, routine clinical follow-up cardiac MRI evaluation of ventricular function and size. Patients were classified into either single left or single right ventricular (SLV and SRV) morphologies as described previously^11^. This study was approved by the Colorado Multiple Institutional Review Board and was part of the Fontan at Altitude Registry for Outcomes study with an approved waiver of informed consent. The study and methods were carried out in accordance with relevant guidelines and regulations including the Declaration of Helsinki.

### Cardiac MRI technique

All patients underwent standardized CMR evaluation per institutional protocol with prescribed sequences customized for the single ventricle evaluation as described previously^9^. Using either 1.5 or 3.0 tesla magnet (Ingenia, Philips Medical Systems, Best, The Netherlands) standard, balanced, ECG-gated steady-state free precession stacks of short-axis images covering the ventricles from base to apex were used for volumetric and functional analysis. In similar fashion, long-axis and 4-chamber views were obtained in each subject with plane orientations detailed to appreciate the atrio-ventricular valves and outflow tracts.

### CMR post-processing

The entire image post-processing was performed using commercially available software Circle CVI42 (version 5.13.19 or higher, Calgary, Canada). Ventricular volumes were derived using the semi-automatic segmentation processing tool of the endocardial contours with subsequent indexing to the body-surface area (BSA). End-diastolic and end-systolic volume indices (EDVi and ESVi) along with EF and cardiac index (CI) were recorded in each patient. Myocardial strain was assessed for both SLV and SRV by drawing endocardial and epicardial contours for the image set within the first phase and automatically propagated throughout the cardiac cycle using the software algorithm. The quality of feature tracking was assessed in each subject in every utilized view. The rudimentary hypoplastic ventricular structure was not consider in this study as a part of the deformation analysis. In this fashion, patient specific strain–time curves were generated and peak GCS and GLS values were sampled. Generated strain–time curves were then exported to MATLAB 2022 (Mathworks, Natick, MA) for further pre-processing and PCA analysis.

### Principal component analysis

**Figure 1:**
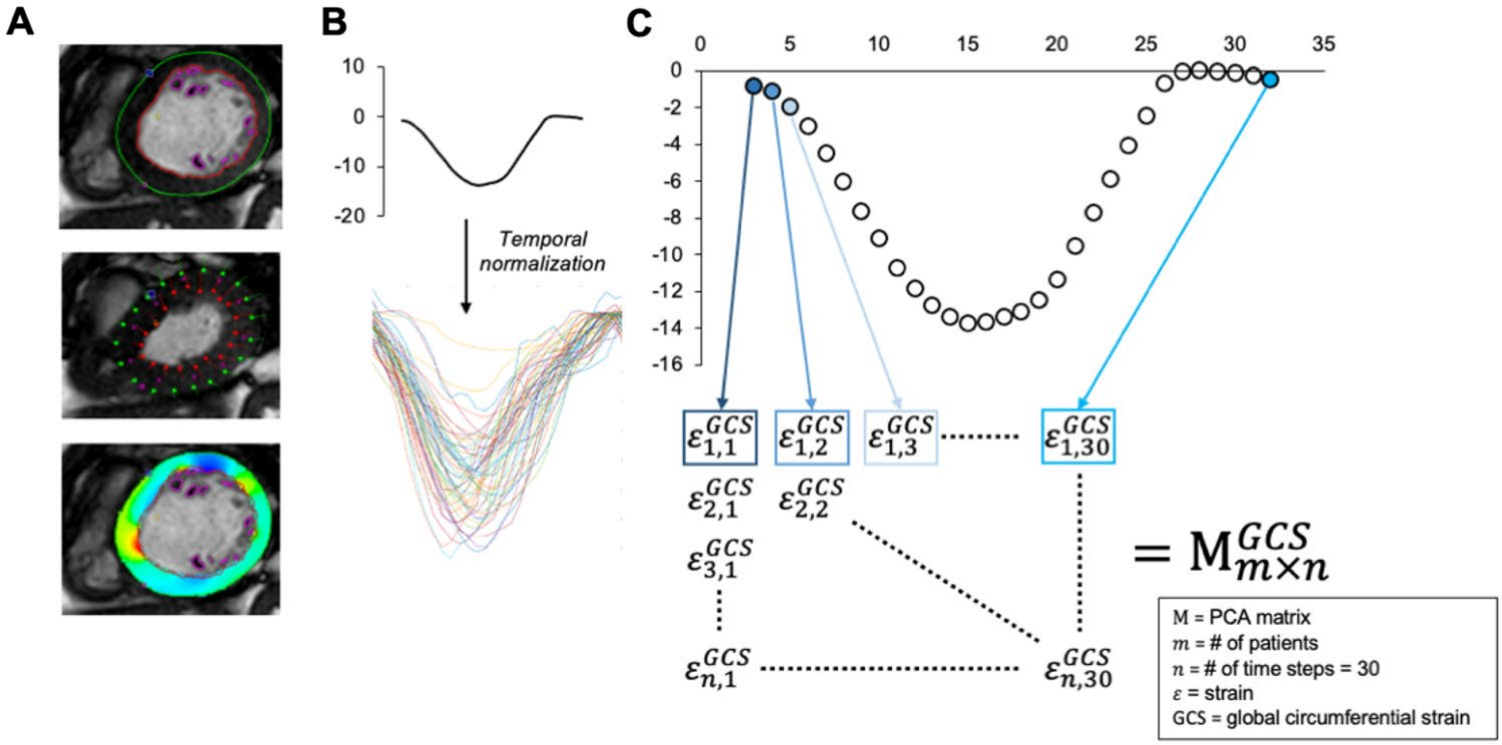
Strain–time curve pre-processing and input into the principal component analysis (PCA). (**A**) After endocardial contouring and generation strain–time curves, (**B**) all signals were temporally adjusted for heart rate and sampling rate. (**C**) Time specific strain values were the iteratively incorporate into the PCA matrix M specific to type of global strain and single ventricle type.

Prior to PCA, individual strain–time curves were temporally normalized to adjust for heart rate variability. The detailed description of this process as well as the PCA work-flow for strain–time signal $\varepsilon \left( t \right)$ is further described in Supplemental material and Graphically in Fig. 1. PCA requires equal-length feature vectors; here we consider each type of global strain (GCS or GLS) signal in a unique analysis. To proceed with temporal normalization, the temporal resolution for each individual subject (CMR derived temporal resolution) was adjusted by the average heart cycle period within the given dataset. This step was motivated by physiologic relationship between increased heart rate (reduction in preload) and lower strain magnitude. To obtain strain type and ventricle morphology specific (SLV or SRV) data matrices with equal length feature vectors, all strain–time curve signals were interpolated to 30 temporal phases (30 time frames per cardiac cycle). This step was required for only small proportion (N = 5) of enrolled subjects as the number of acquired SSFP phases is typically pre-set by our institutional protocol to 30. The final temporarily and time-step normalized strain–time curves were entered into PCA matrices specific to (1) either GCS or GLS and (2) SLV and SRV group.

In mathematical terms, PCA uses an orthogonal transformation to convert a set of potentially correlated variables [strain value at individual cardiac phase—$\varepsilon \left( 1 \right)$, $\varepsilon \left( 2 \right)$, $\varepsilon \left( 3 \right)$, …, $\varepsilon \left( {30} \right)$] into a set of new, linearly uncorrelated vectors, known as principal components (PCs). In other words, PCA seeks to identify underlying patterns in the collected set of ventricular strain–time curves and describes them in terms of a smaller number of parameters (PCs and their corresponding variances). In the context of this study, the calculated PCs represent modulators altering the shape of strain–time curve. Additionally, PCs are accompanied by their corresponding set of score values representing the resemblance between the patient specific strain–time curve and observed PC.

These curve-based shape scores are scalar values representing a dot product between considered PC vector **p**_n_ and patient-specific strain-curve vector. In other words, this score describes how much does each individual patient strain pattern resemble a specific PC vector. Exact mathematical expression is as follows:

$$PC score_{n}^{i} = {\varvec{p}}_{{\varvec{n}}} \cdot \varepsilon_{i}$$

where **p**_n_ represents specific principal component vector and $n \in \left\{ {1,30} \right\}$ and ${\varvec{\varepsilon}}_{i}$ represents a patient specific strain-curve vector with $i \in \left\{ {1,55} \right\}$ for single right ventricle patients and $i \in \left\{ {1,67} \right\}$ for single left ventricle patients. Only PCs cumulatively accounting for > 90% of explained variation (set of first three PCs) were included for comparative and correlative analyses.

### Statistical analysis

All statistical analyses and data presentation were performed with SAS (SAS Analytical Software) and Prism (version 9.0 or higher, GraphPad Software Inc, La Jolla, Calif). Variables were checked for the distributional assumption of normality using normal plots, in addition to D’Agostino-Pearson, Shapiro–Wilk, and Kolmogorov–Smirnov tests. Variables that were skewed were natural log transformed. Demographic and clinical characteristics are summarized with descriptive statistics (mean and standard deviation, median and 25th/75th percentiles, frequencies and percentages). Variables that were positively skewed (e.g. end-diastolic and systolic volume indexes) were natural log transformed for the correlative analyses. Demographic and clinical characteristics among SLV and SRV patients were compared using the unpaired *t*-tests, Mann–Whitney test, chi-square, or Fisher’s exact tests as dictated by the nature of sampled variables.

Single variable Cox proportional hazard analysis was applied to assess the predictive ability separately in SLV and SRV groups. The composite outcome reflective of Fontan related complications was defined as death, transplantation, development of plastic bronchitis, and protein losing enteropathy. All patients were followed up to the particular event or to the end of the study (December 2022). For variables that were found to be significantly associated with survival univariable analysis, Kaplan–Meier survival curves were constructed with the specific cut-off values derived from receiver operating characteristic to construct binary group variables. The survival between the two groups was then compared using the log-rank test. Performed analyses were considered exploratory and hypothesis generating and adjustments for multiple comparisons were not employed. All performed tests were 2-sided and significance was based on an alpha-level of ≤ 0.05.

## Results

### Patient characteristics and standard CMR hemodynamics

A total of 122 patients with Fontan circulation, out of which 67 were classified into SLV and 55 into SRV categories, were included in the study. Specific diagnoses included tricuspid atresia (n = 43), hypoplastic left heart syndrome (n = 41), double outlet right ventricle (n = 12), double inlet left ventricle (n = 9), unbalanced AV canal (n = 6), pulmonary atresia with intact ventricular septum (n = 7), tricuspid stenosis/pulmonary stenosis (n = 3), and crisscross heart (n = 1). The demographic and standard CMR hemodynamics are summarized in Table 1. The mean age at Fontan operation was 3.4 ± 1.1 years and mean age at time of CMR of acquisition was 12.7 ± 4.7 years. The predominant type of Fontan operation was extra-cardiac conduit (n = 103, 84%). At the time of CMR acquisition, all patients were in regular sinus rhythm. In general, in comparison to SLV group, patients with SRV morphology were younger at the time of CMR and had higher EDVi and ESVi, reduced EF, as well as decreased peak GCS and GLS. Within the SRV group, 4 patients had moderate plus degree of the atrioventricular valve regurgitation as assessed by echocardiography, other had trivial-to-mild degree of severity.

**Table 1:** Patient Characteristics and CMR Hemodynamics.

	All Patients (N = 122)	SLV (N = 67)	SRV (N = 55)	*p*-value
Age at Fontan (years)	3.4 ± 1.1	3.5 ± 1.2	3.4 ± 0.9	0.643
Age at CMR (years)	12.7 ± 4.7	14.4 ± 4.1	10.6 ± 4.4	< 0.001
Sex (Female %)	54 (44.2%)	35 (52.2)	19 (34.5)	0.076
BSA (m^2^)	1.4 ± 0.4	1.5 ± 0.3	1.2 ± 0.4	< 0.001
Lateral tunnel	19 (15.6%)	9 (13.4%)	10 (18.2%)	0.614
Extra cardiac conduit	103 (84.4%)	58 (86.6%)	45 (81.8%)	0.639
EDVi (ml/m^2^)	91 (74—112)	81 (67—96)	101 (88—131)	< 0.001
ESVi (ml/m^2^)	47 (34—60)	39 (31—52)	54 (45—75)	< 0.001
EF (%)	48 ± 8	51 ± 7	45 ± 9	< 0.001
CI (L/min/m^2^)	3.5 ± 1.3	3.2 ± 1.0	3.9 ± 1.5	0.003
Heart rate	77 ± 20	76 ± 16	77 ± 25	0.758
Peak GCS (%)	− 13.5 ± 3.5	− 15.4 ± 3.0	− 11.1 ± 2.6	< 0.001
Peak GLS (%)	− 12.6 ± 3.1	− 13.4 ± 3	− 11.7 ± 2.8	0.002

All data reported as mean ± SD or as median with corresponding IQR.

*CMR* cardiac magnetic resonance, *BSA* body surface area, *EDVi* end-diastolic volume index, *ESVi* end-systolic volume index, *EF* ejection fraction, *CI* cardiac index, *GCS* global circumferential strain, *GLS* global longitudinal strain. *p*-values represent unpaired *t*-test or Mann–Whitney test.

**Figure 2:**
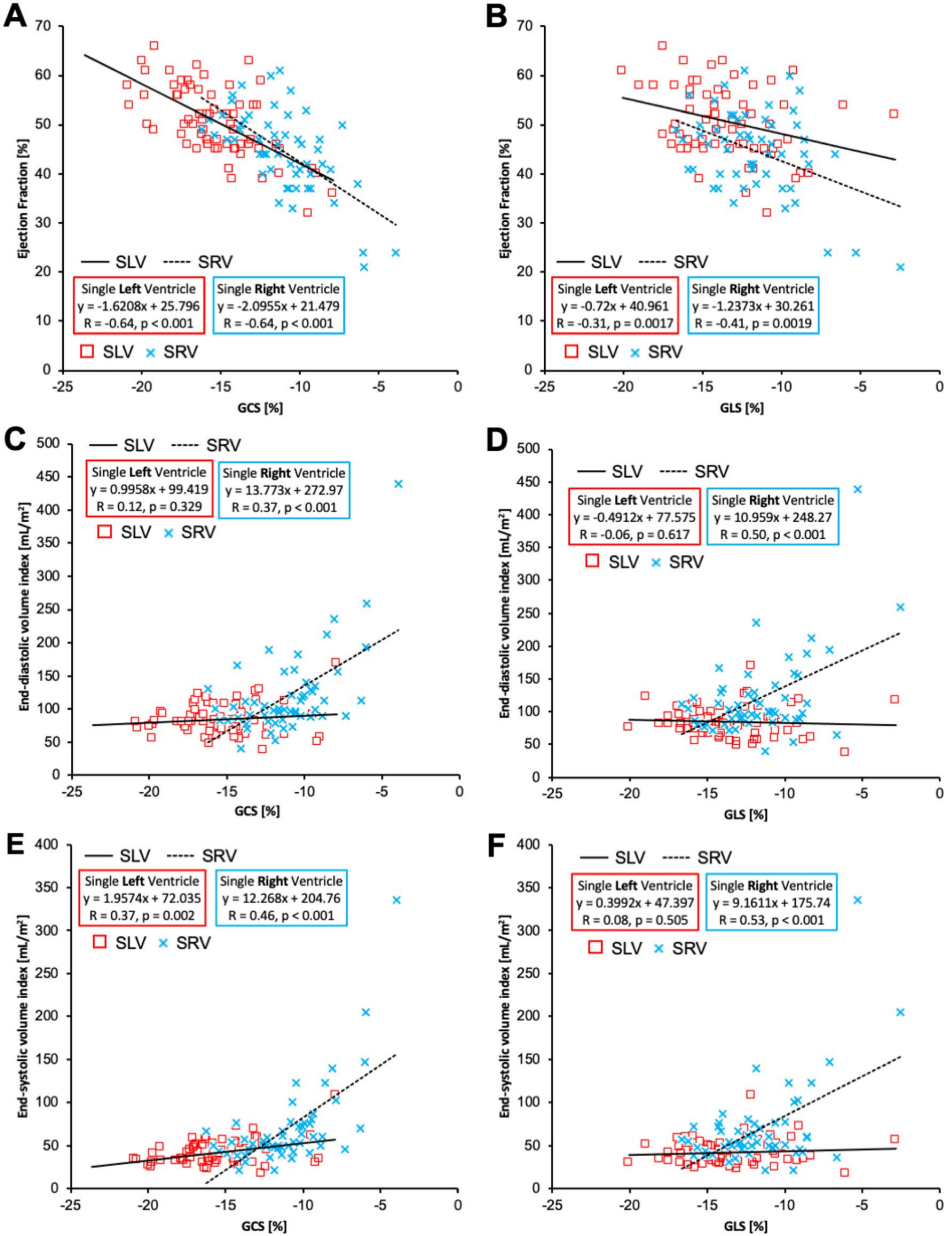
Relationship between peak strain indices and standard CMR measures of ventricular size and function. (**A**) GCS correlated with ejection fraction in similar manner for both SLV and SRV groups with higher *d*EF/*d*GCS or steeper slope for SRV group. (**B**) GLS was associated with EF for both SLV and SRV groups but to a lesser degree than GCS, again with higher *d*EF/*d*GCS in SRV patients. (**C**) GCS was not associated with EDVi in SLV group but lower GCS values were associated with higher EDVi in SRV group. (**D**) Similarly, GLS was not associated with EDVi in SLV group, but reduced GLS was associated with higher EDVi in SRV patients. (**E**) GCS was associated with ESVi in both SLV and SRV groups with higher dESVi/dGCS in SRV group. (**F**) GLS did not reveal any relationship with ESVi in SLV group but showed a significant relationship in SRV group.

The relationship between peak strain variables and standard CMR volumetric and function indices is depicted in Fig. 2. Peak GCS correlated with ejection fraction in similar manner for both SLV (r = − 0.64, *p* < 0.001) and SRV (r = − 0.64, *p* < 0.001) groups with steeper slope encountered in the SRV group (SRV: *d*EF/*d*GCS ~ − 2.1, SLV: *d*EF/*d*GCS ~ − 1.6). To a lesser degree, peak GLS was associated with EF for both SLV (r = − 0.31, p = 0.002) and SRV (r = − 0.41, *p* = 0.002) groups again with higher slope observed in SRV patients (SRV: *d*EF/*d*GLS ~ − 1.2, SLV: *d*EF/*d*GLS ~ − 0.7). Peak GCS was not associated with EDVi in SLV group but revealed positive trend in SRV group (r = 0.37, *p* < 0.001) indicating decreased strain in more dilated ventricle. Correspondingly, GLS was not associated with EDVi in SLV group, but reduced GLS was associated with higher EDVi in SRV patients (r = 0.50, *p* < 0.001). GCS was associated with ESVi in both SLV (r = 0.37, *p* = 0.002) and SRV (r = 0.46, *p* < 0.001) groups with higher slope observed in SRV group (SRV: *d*ESVi/*d*GCS ~ 12.3, SLV: *d*ESVi/*d*GCS ~ 2.0). Peak GLS did not reveal any relationship with ESVi in SLV group but showed a significant relationship in SRV group (r = 0.53, *p* < 0.001).

### Strain–time curve morphology

**Figure 3:**
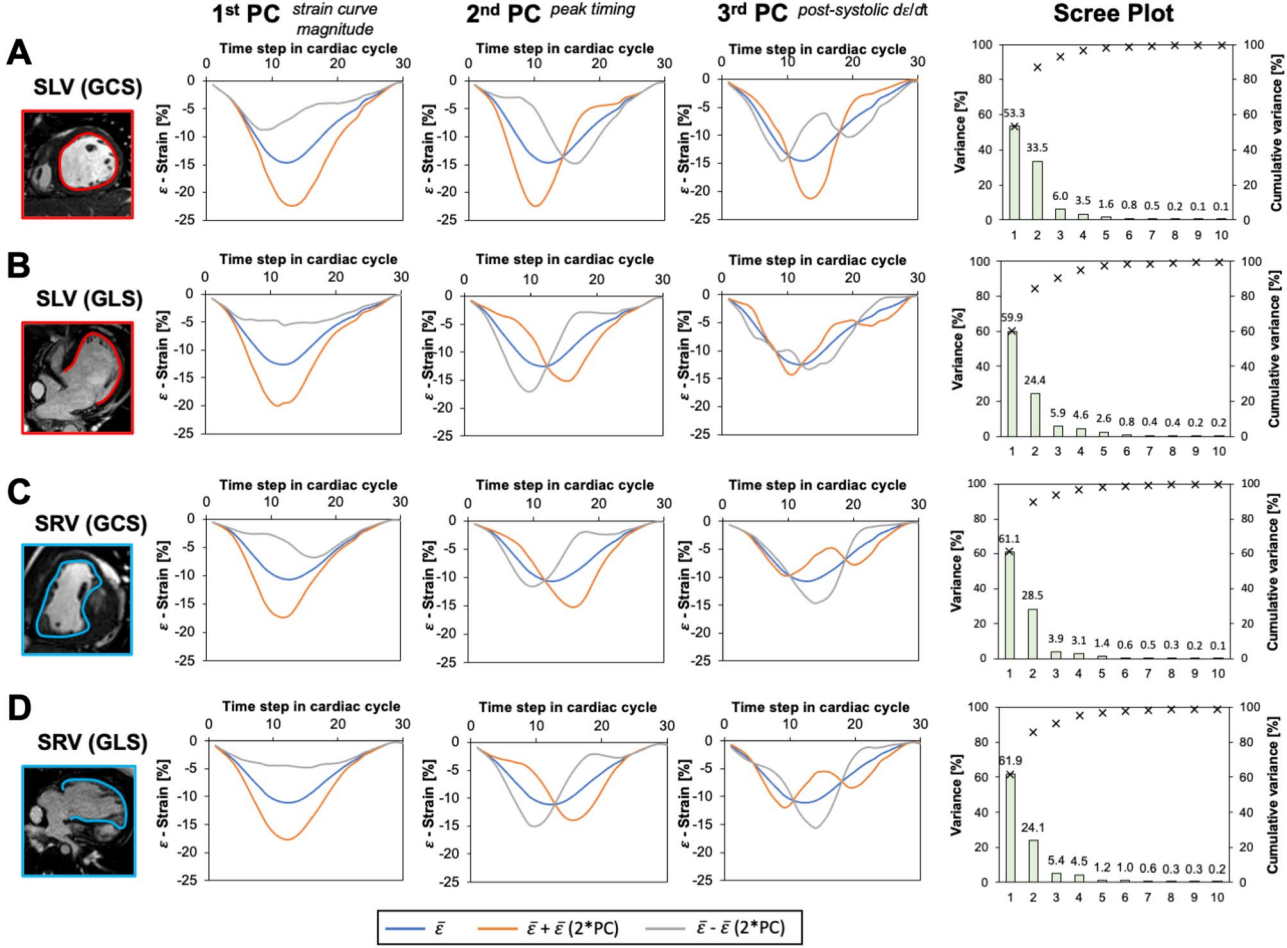
Graphical summary of the strain–time principal component analysis. Principal components derived for the SLV patients specific or global circumferential strain (**A**) and global longitudinal strain (**B**) revealed similar modes of deformation with similar proportion of individual components accounting for the cumulative variance. Similar observation was noted in the SRV group for GCC (**C**) and GLS (**D**) strain–time curves. *SLV* single left ventricle, *SRV* single right ventricle, *GCS* global circumferential strain, *GLS* global longitudinal strain, *PC* principal component, $\overline{\varepsilon }$ = mean strain–time curve.

**Figure 4:**
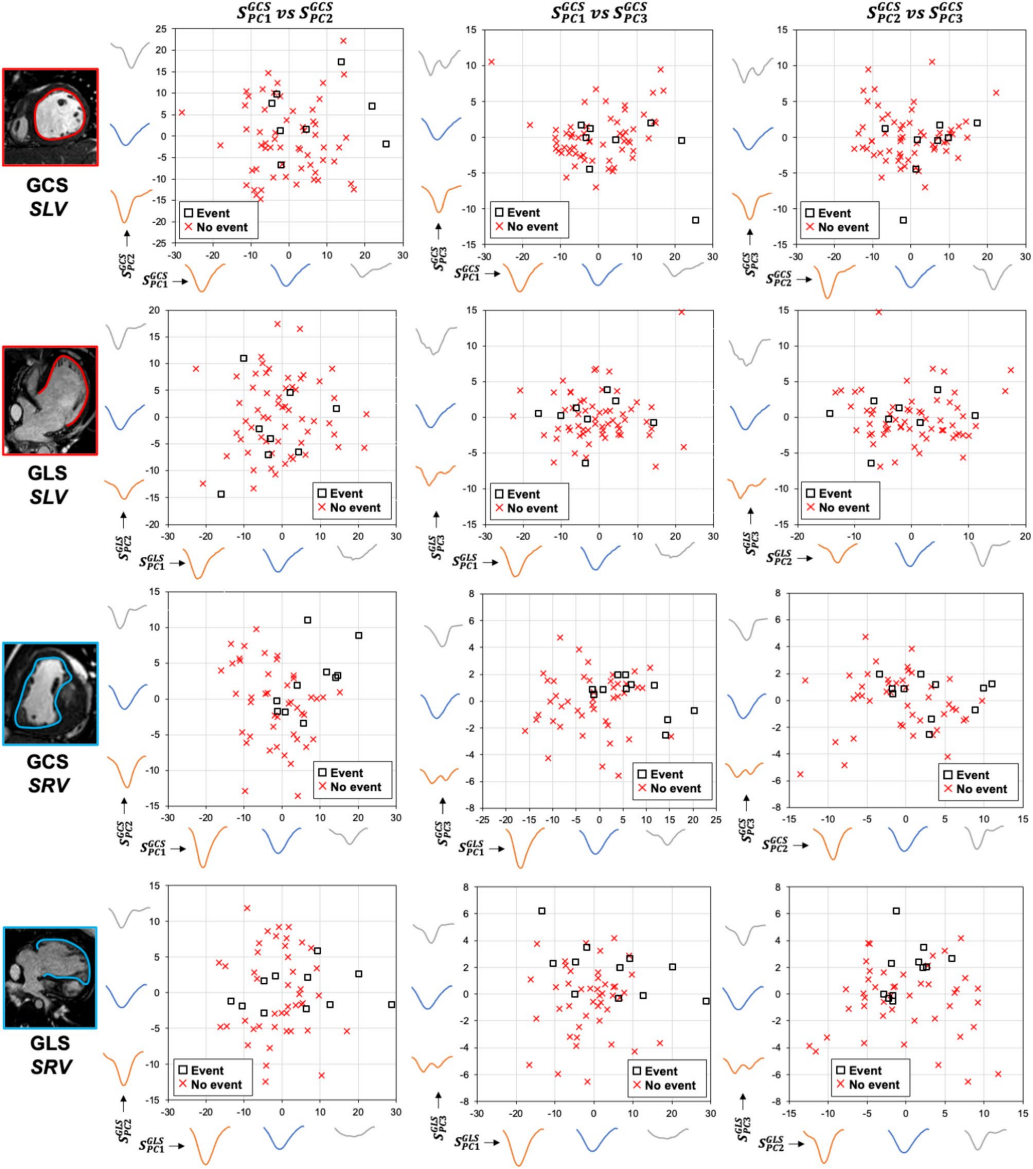
The combination of the first three PCs strain scores plotted against each other for specific and single ventricle morphology and type of strain. X- and Y- axes further display how are calculated strain–time curve scores reflective of shape deformation. *SLV* single left ventricle, *SRV* single right ventricle, *GCS* global circumferential strain, *GLS* global longitudinal strain, *PC* principal component, *S* = principal component specific score individual to each patient.

Figure 3 summarizes the PCA results for each individual patient group and the type of strain. The first three PC describing strain–time curve variations accounted for > 90% of total variation in each analysis and hence were considered for further analysis. Features described by PCs were similar regardless of group and type of strain. To appreciate the effect of PCs on strain–time curve, we plotted mean strain ($\overline{\varepsilon })$ curve (blue line) for specific type of strain and single ventricle group and artificially created extreme variations of this mode in both positive (orange line) and negative (grey line) directions. Scree plots for each combination of strain type—single ventricle morphology depict specific PC contribution to the overall variability in each dataset. The first PC accounted for > 50% of variation in all datasets, universally described variation in strain–time curve peak height, hence strain magnitude. The second PC accounting approximately for ~ 25% of variation in each dataset, described time-to-peak variability of strain–time curve. Lastly, the third PC accounting for ~ 5% variation in each dataset, described variation in post-systolic slope *d*$\varepsilon$/*d*t of the strain–time curve. To graphically represent the interaction of the first three PCs and their association with the clinical outcomes (event vs no event) we plotted a combination of patient specific PC scores on each other with superimposed labels describing their event status (Fig. 4).

### Clinical outcomes

The median time to follow up was 2.1 years (IQR 0.9–4.5 years). In this time, 8 events were encountered in the SLV group including heart transplant (n = 3), referral for heart transplant evaluation (n = 2), death (n = 2), and plastic bronchitis (n = 1). Regarding the SRV patient group, 11 events occurred including heart transplant (n = 5), death (n = 4), plastic bronchitis (n = 1), and protein losing enteropathy (n = 1). Cumulative freedom from the clinical events for all patients was 94.2% at 1-year and 90.5 at 2-years. There was no difference in freedom from events between SLV and SRV groups (log-rank *p* = 0.123). The comparison of MRI hemodynamics and strain parameters between patient with and without events is portrayed in Table 2.

**Table 2:** Cardiac MRI Hemodynamics.

	Single left ventricle			Single right ventricle		
	No event (N = 59)	Event (N = 8)	*p*-value	No event (N = 44)	Event (N = 11)	*p*-value
EDVi (ml/m^2^)	81 (67–96)	85 (68–105)	0.723	99 (84–121)	156 (96–212)	0.017
ESVi (ml/m^2^)	39 (32–50)	41 (30–62)	0.579	52 (43–66)	72 (50–147)	0.020
EF (%)	51 ± 7	48 ± 10	0.386	46 ± 7	39 ± 13	0.128
CI (L/min/m^2^)	3.2 ± 1.0	3.3 ± 1.0	0.704	3.8 ± 1.2	4.6 ± 2.2	0.263
Heart rate	76 ± 16	80 ± 16	0.493	75 ± 23	79 ± 26	0.622
Peak GCS (%)	− 15.8 ± 2.8	− 12.9 ± 3.0	0.029	− 11.8 ± 2.2	− 8.4 ± 2.4	< 0.001
GCS PC1 score	0.9 ± 10.4	6.7 ± 12.1	0.128	− 1.8 ± 7.4	7.3 ± 7.0	0.001
GCS PC2 score	− 0.6 ± 8.5	4.5 ± 7.5	0.104	− 0.8 ± 5.5	3.1 ± 4.9	0.034
GCS PC3 score	0.2 ± 3.5	− 1.4 ± 4.6	0.352	− 0.1 ± 2.2	0.4 ± 1.4	0.334
Peak GLS (%)	− 13.4 ± 3.2	− 13.4 ± 2.3	0.984	− 12.1 ± 2.2	− 9.9 ± 4.1	0.116
GLS PC1 score	− 1.0 ± 7.2	1.5 ± 5.2	0.255	− 1.1 ± 7.4	4.4 ± 13.0	0.200
GLS PC2 score	− 0.4 ± 6.1	− 0.3 ± 1.6	0.923	− 0.1 ± 6.1	0.3 ± 2.8	0.785
GLS PC3 score	0.3 ± 3.1	− 0.1 ± 0.3	0.331	− 0.5 ± 2.6	1.8 ± 2.0	0.005

All data reported as mean ± SD or as median with corresponding IQR.

*EDVi* end-diastolic volume index, *ESVi* end-systolic volume index, *EF* ejection fraction, *CI* cardiac index, *GCS* global circumferential strain, *GLS* global longitudinal strain, *PC* principal compoent. *p*-values represent unpaired *t*-test or Mann–Whitney test.

**Figure 5:**
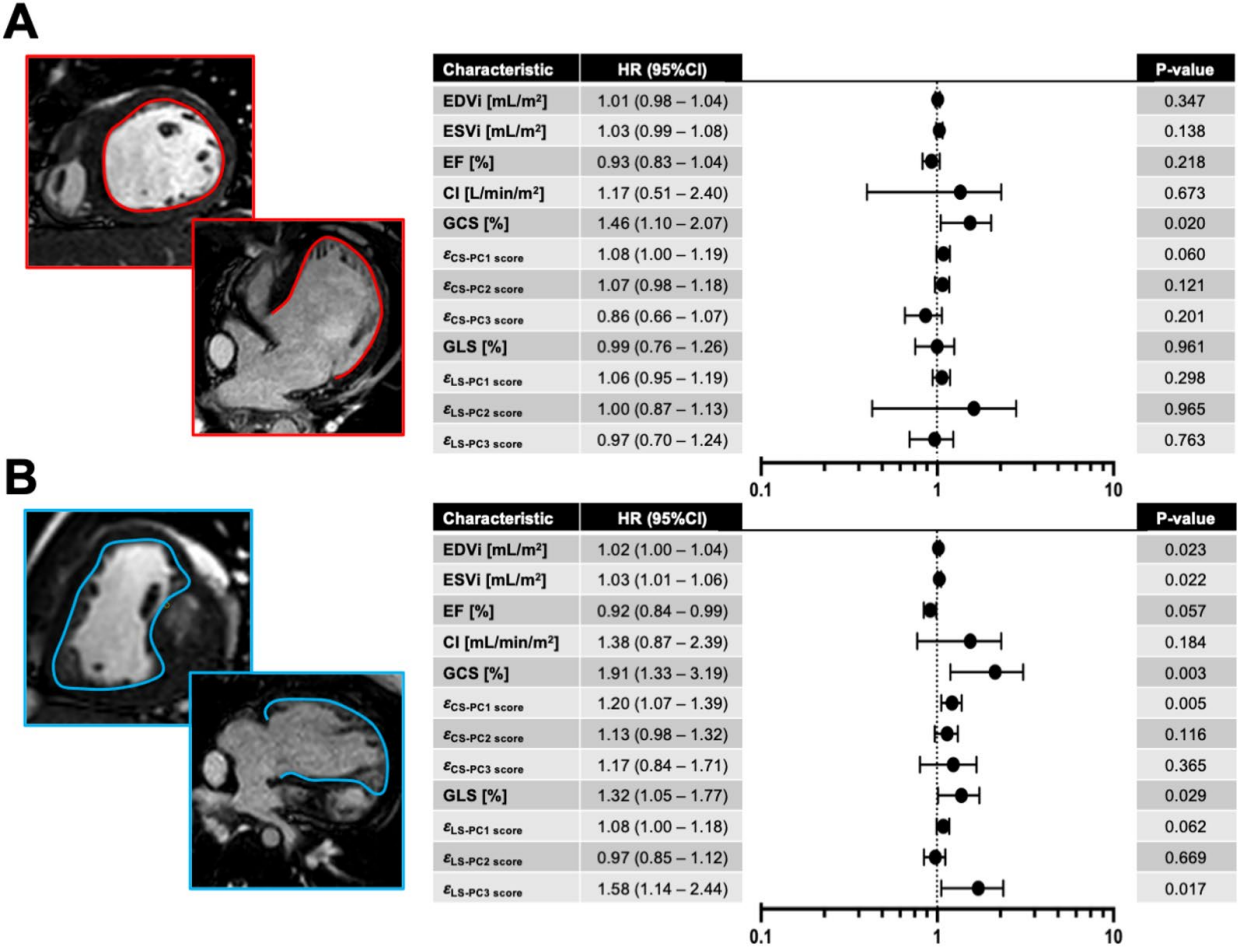
Forest plot summarizing single variable Cox proportional hazard analysis for standard CMR and myocardial strain markers. (**A**) Single left ventricle specific plot highlighting GCS as the only predictor of composite clinical events. (**B**) Single right ventricle plot depicting EDVi, ESVi. GCS, $\varepsilon_{CS - PC1}$, GLS, and $\varepsilon_{LS - PC3}$ as markers associated with clinical events. *GCS* global longitudinal strain, *GLS* global longitudinal strain, *EDVi* end-diastolic volume index, *ESVi* end-systolic volume index, *EF* ejection fraction, $\varepsilon$ = strain (LS/CS longitudinal or circumferential strain), *PC* principal component, *HR* hazard ratio.

**Figure 6:**
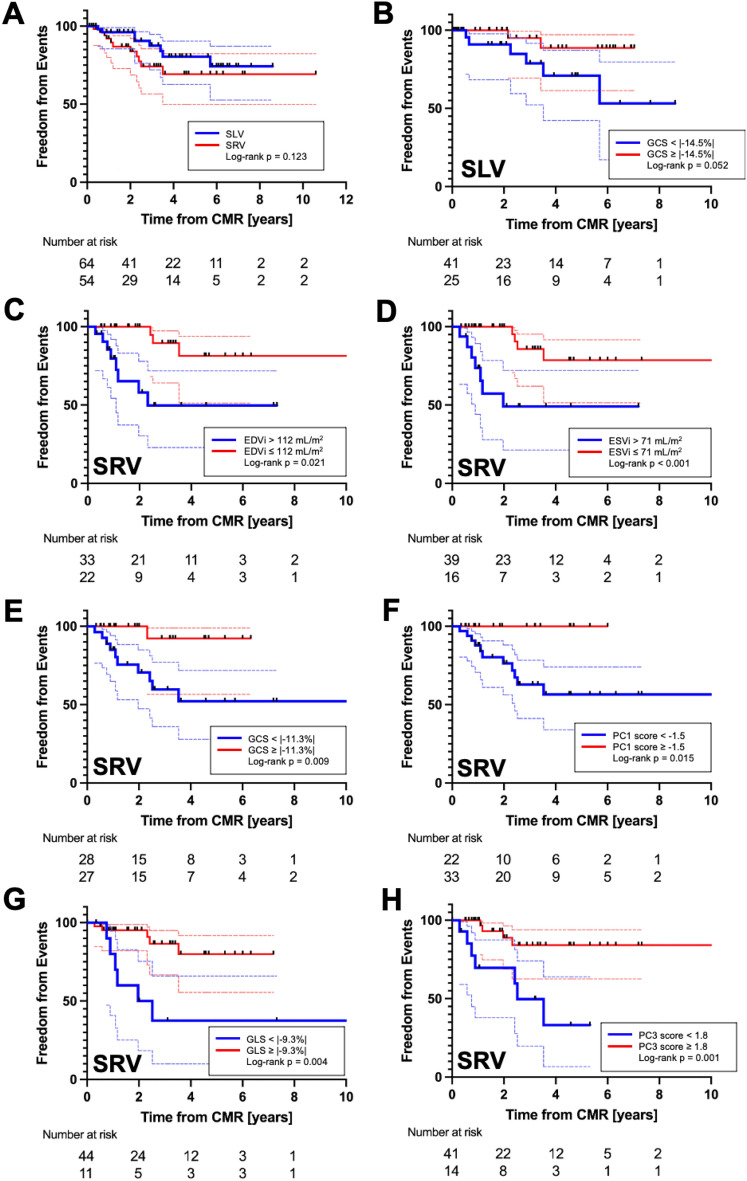
Kaplan–Meier plots for SLV versus SRV comparison (**A**) and variables found to be predictive of clinical events on single variable Cox proportional hazard analysis. (**B**) GCS measured in the SLV group with cutoff value 14.5% approached statistical significance. In the SRV group, (**C**) EDVi > 112 mL/m^2^ and (**D**) ESVi > 71 mL/m^2^ had worse freedom from clinical events. (**E**) GCS in the SRV patients with value worse than − 11.3% and its related first PC score < − 1.5 (**F**) had overall worse freedom from events. GLS measured in SRV patients worse than − 9.3% (**G**) and its related third PC score < 1.8 (**H**) were also associated with cumulatively worse outcomes.

The summary of univariable proportional hazard analysis is depicted in Fig. 5. Considering the SLV group, the only variable associated with clinical events was the GCS (HR = 1.46, *p* = 0.020). Regarding the SRV group, variables associated with clinical events were EDVi (HR = 1.02, *p* = 0.023), ESVi (HR = 1.03, *p* = 0.022), GCS (HR 1.91, *p* = 0.003), GCS derived PC1 score (HR 1.20, *p* = 0.005), GLS (HR 1.32, *p* = 0.029), and GLS derived PC3 score (HR 1.58, *p* = 0.017). Subsequent Kaplan–Meier analysis applied to all variables predicting clinical events is portrayed in Fig. 6. For patients in SLV groups, the ROC analysis provided an optimal GCS value of 14.5% (absolute value) to define groups with different freedom from clinical events approaching statistical significance (*p* = 0.052). Considering the SRV patients, EDVi > 112 mL/m^2^ and ESVi > 71 mL/m^2^ defined subgroups of patients with worse cumulative freedom from events (*p* = 0.021 and *p* < 0.001, respectively). Applying the GCS measures to the SRV group, patient subgroups with peak GCS < 11.3% (absolute value) and its associated first PC score < − 1.5 were predictive of decreased freedom from events (*p* = 0.009 and *p* = 0.015, respectively). Lastly, using the GLS measures in the SRV group, patient subgroups with peak GLS < 9.3% (absolute value) and its associated third PC score < 1.8 were predictive of decreased freedom from events (*p* = 0.004 and *p* = 0.001, respectively).

## Discussion

Myocardial performance evaluation by strain analysis derived by CMR feature tracking is increasingly utilized method in patients with Fontan circulation^1,12^. In this study we performed detailed myocardial strain analysis separately in SLV and SRV patients late after Fontan operation to better understand contribution and patterns of myocardial deformation to overall single ventricle function. We observed that GCS and GLS contribute differently to EF in SLV and SRV patients, and their associations with ventricular size parameters vary as well. Using PCA, we further explored the different patterns of strain–time curves and described distinct curve shapes, some of which are strongly associated with clinical events. In general, strain derived parameters performed better than EF and ventricular size indices in predicting clinical events individually in both SLV and SRV patients. Our findings align with previous results in morphological normal hearts^6^ and single ventricle studies^2,13^ demonstrating strain as a more sensitive marker of subclinical systolic dysfunction.

Significant differences in systemic ventricular morphologies exist in extrinsic geometric properties but also at the intrinsic tissue level, as dictated by embryologic origins. Right and left ventricular myocardial substrate differs in myocardial fiber orientation, myocyte size and contractile properties, matrix composition, and even myocyte cellular metabolism^14,15^. Consequently, adaptation to adverse loading conditions is different as well^16^. In patients with SRV morphology, the myocardial strain patterns are dynamically changing during^17^ and also after single ventricle palliation^13^. In morphologically normal right ventricle, GLS is the predominant mode of global strain, even in patients with pulmonary hypertension with a GCS/GLS ratio < 1^18,19^. In patients with hypoplastic left heart syndrome and its pathologic variants, the GCS/GLS ratio can approach 1.0 and can fully reverse in some patients late after Fontan operation^1,2,13^. In our study, we showed that *d*EF/*d*GCS slope is steeper than *d*EF/*d*GLS slope in SRV patients, suggesting that circumferential strain is a larger contributor to overall pump function. Interestingly, the *d*EF/*d*GCS was steeper in SRV than in SLV patients. Previous studies have suggested that chronic remodeling of SRV to a more primary circumferential mode of strain to resemble morphologically normal left ventricle might generate more contractile power^17^. However, it is not known to a what extent is this remodeling adaptive or maladaptive.

Only a limited number of studies have investigated the global strain patterns in relation to myocardial function^7^. Segment specific strain–time curves are more typically qualitatively and quantitatively evaluated for the purposes of evaluating intraventricular mechanical dyssynchrony or discoordination^20–22^. In this study we applied an unbiased mathematical technique to distinguish primary modes of strain–time curve variances. We systematically explored the effect of calculated PCs on average strain-curve shape for a specific single ventricle morphology group and for a specific type of global strain. Per our results, strain–time curve morphologies and their variations are very similar regardless of ventricular morphology or global strain type. As expected, the most prominent variation in strain–time curve is variability in overall amplitude of the signal, translating to standard clinical measurements of peak GCS or GLS. This observation could be effectively eliminated from the results by normalizing all strain–time curves to height prior to PCA, but might ultimately eliminate interesting interactions with other PCs.

The second PC described the early versus late onset of peak strain values which are typically quantified in strain analyses as time-to-peak measurements. From our study it is not clear whether early or late strain peak onset is beneficial in either SLV or SRV groups as there was no association between the second PC and clinical outcomes. Unfortunately, time-to-peak measurements are typically reserved for myocardial segment specific analyses for strain based mechanical dyssynchrony and global strain time-to-peak values are typically not evaluated. Theoretically, the reasonable correlate to global time-to-peak strain would be systolic-to-diastolic time ratio typically derived from the tissue Doppler wave^23^. High time-to-peak strain and systolic-to-diastolic time ratio values are typically associated with heart failure progression and overall worse clinical outcomes regardless of ventricular morphology^24,25^. Systolic prolongation and hence the second PC score might be also impacted by higher post-systolic strain and mechanical dyssynchrony which we did not consider in this study.

**Figure 7:**
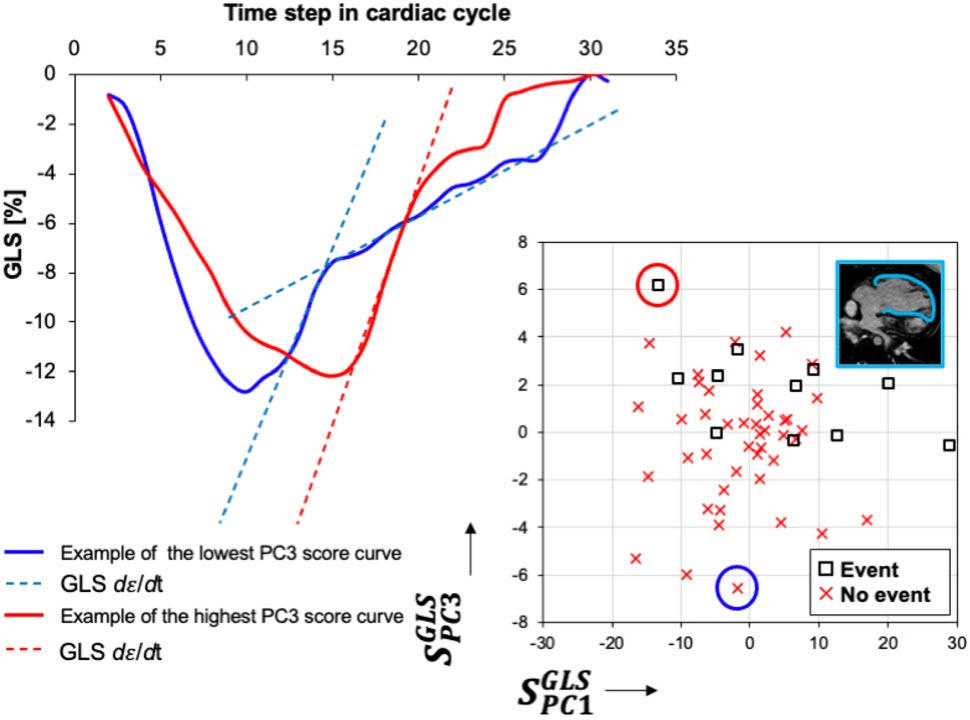
Graphical demonstration of the third principal component describing the post-systolic *d*$\varepsilon$/*d*t pattern derived from GLS curves in patients with SRV morphology. Strain–time curves of the two most representative patients with the most extreme opposite score values are depicted. Blue strain–time curve depicts a patient without clinical event demonstrating two distinct post-systolic relaxation slopes in early and late diastole, respectively (blue-dashed lines). This is analogous to a double peak diastolic strain rate pattern conventionally described as early and late diastolic strain rates. On the opposite end of the spectrum is a patient with a clinical event and corresponding strain–time curve (red) with primarily a single *d*$\varepsilon$/*d*t slope throughout the majority of diastole (single dashed red line). This would translate to a single peak diastolic strain rate.

The third PC primarily described the post-systolic strain–time curve morphology, specifically variation in post-peak *d*$\varepsilon$/*d*t from unified to more dynamic negative slope pattern. A more dynamic *d*$\varepsilon$/*d*t pattern would be seen on standard strain rate curve analysis as a double diastolic peak, with an early and late diastolic strain rate peak. Interestingly, SRV patients with a more complex diastolic *d*$\varepsilon$/*d*t pattern derived by GLS, reflective of prominent late diastolic strain rate peak were less likely to develop a clinical event (Fig. 7). To our knowledge, the significance of late diastolic strain rate is yet to be determined. Rumbinaite et al. showed that low-dose dobutamine stress test increases late diastolic strain rate in patients with no evidence of coronary artery disease but fails to show any change in patients with coronary disease^26^. This beneficial effect of the late diastolic strain rate is indirectly supported in our results as patients with higher scores reflective of minimal or no late diastolic strain rate were more likely to have a clinical event. We did not purposefully work with strain rate curves as these values would be subjected to sampling error when derived from CMR using our acquisition parameters. We hope that we will be able to validate this finding in future studies or using echocardiography derived strain and strain rate curves.

The utility of CMR derived global strain based measurements to predict functional worsening and clinical outcomes long after successful Fontan palliation and clinical outcomes has been emphasized in previous studies^2,12,27^. In our study, GCS is the only CMR marker associated with cardiac events in patients with SLV. For SRV patients, both GLS and GCS were predictive of clinical with GCS values, showing the strongest potential on univariate hazard analysis. GCS was shown to be more associated with transplant-free survival than GLS in what is currently the largest CMR based Fontan study^1^. The authors identified using classification and regression tree analysis that GCS values worse than -6.6% to be highly predictive of death or transplant including both SLV and SRV patients into the analysis. In our group specific study, GCS cut-off values were − 14.5% and − 9.3% for SLV and SRV groups, respectively, acknowledging the that our composite outcome included non-terminal Fontan physiology related complications including protein losing enteropathy and plastic bronchitis. Mechanistically, global strain values are better at detecting subclinical systolic dysfunction than EF, which is confounded by ventricular size and can be maintained in normal range by increasing ventricular wall thickness. Additionally, global strain based measurements are now easily obtainable from many commercially available post-processing tools for both CMR and echocardiography with data suggesting more reproducible values than EF^28^.

### Study limitations

Limitations to our study are primarily inherent to a single center retrospective cohort study. Upon further division of patients based on SLV and SRV morphologies the small number of clinical end-points limited any form of multivariable predictive analysis. Therefore, important demographic covariates previously shown to be associated with Fontan outcomes including sex, age, and time from Fontan operation were not included^29^. This is further pertinent to likely type II error in our analysis when comparing CMR markers between patients with and without clinical end-points. In comparison to some previous studies investigating myocardial strain in single ventricle patients^1,2^, we did not perform endocardial segmentation to include both dominant and the hypoplastic ventricle. This decision was motivated by premise to input PCA matrix with strain–time signal reflective of not confounded by highly variable anatomic remnant structures. Our reported median GLS and GCS values are very similar to those reported in previous large study applying the identical post-processing pipeline and software platform^1^. However, we acknowledge that considering the hypoplastic structures is important from the perspective of overall mechanical efficiency and dyssynchrony. Our study also included acquisitions from 1.5 and 3.0 T field strength systems but intra-observer variability in feature tracking strain at 3.0 T is similar to that at 1.5 T^30^. Future studies will focus on whether myocardial segment specific strain and type of endocardial segmentation impacts the PCA and predictive analysis. Important catheterization-derived hemodynamics and were not available in majority of patients due to lack of clinical indication or large time period between the catheterization study and CMR. Additionally, we plan to study strain-based markers including the strain–time curve morphology in association with other imaging biomarkers of Fontan complications such as elastography derived liver stiffness and cardiopulmonary exercise capacity. Furthermore, to make PC scores reflective of strain curve morphology clinically more available, we plan to develop an online strain curve repository tool from which PC model can be developed to yield patient specific PC scores for wide application and validation of our results.

## Conclusions

CMR derived global strain measures are sensitive markers of clinical outcomes in patients with Fontan circulation, particularly in patient with the SRV morphology. These results support ongoing motion to replace EF as a measurement of myocardial function even in patients with complex single ventricle anatomy. The newly introduced concept of myocardial strain–time curve morphology showed that primary strain shape variations in GCS and GLS are similar between SLV and SRV patients. Our results show promise that quantitative description of the strain–time curve morphology inspired by unbiased PCA technique can predict clinical outcomes and serve as additional marker of single ventricle dysfunction.

## Supplementary Information

Supplementary Information.

## Data Availability

This study has the appropriate research ethics committee approval of COMIRB (Colorado Mutli-institutional Review Board). The data are available as requested from the corresponding author Dr. Michal Schäfer.

## References

[1] Meyer SL, St. Clair N, Powell AJ, Geva T, Rathod RH (2021). Integrated clinical and magnetic resonance imaging assessments late after Fontan operation. J. Am. Coll. Cardiol..

[2] Meyer SL (2020). Serial cardiovascular magnetic resonance feature tracking indicates early worsening of cardiac function in Fontan patients. Int. J. Cardiol..

[3] Dardeer AM, Hudsmith L, Wesolowski R, Clift P, Steeds RP (2018). The potential role of feature tracking in adult congenital heart disease: Advantages and disadvantages in measuring myocardial deformation by cardiovascular magnetic resonance. J. Congenit. Cardiol..

[4] Kato A (2017). Pediatric Fontan patients are at risk for myocardial fibrotic remodeling and dysfunction. Int. J. Cardiol..

[5] Rösner A (2018). Classic-pattern dyssynchrony in adolescents and adults with a Fontan circulation. J. Am. Soc. Echocardiogr..

[6] Stokke TM (2017). Geometry as a confounder when assessing ventricular systolic function: Comparison between ejection fraction and strain. J. Am. Coll. Cardiol..

[7] Badagliacca R (2021). Right ventricular strain curve morphology and outcome in idiopathic pulmonary arterial hypertension. Cardiovasc. Imaging.

[8] Fudim M (2017). The prognostic value of diastolic and systolic mechanical left ventricular dyssynchrony among patients with coronary heart disease. J. Am. Coll. Cardiol..

[9] Schäfer M (2020). Flow profile characteristics in Fontan circulation are associated with the single ventricle dilation and function: Principal component analysis study. Am. J. Physiol. Heart Circ. Physiol..

[10] Ferrari MR, Schäfer M, Hunter KS, Di Maria MV (2023). Coupled waveform patterns in the arterial and venous Fontan circulation are related to parameters of pulmonary, lymphatic and cardiac function. Int. J. Cardiol. Congenit. Hear. Dis..

[11] Anderson PAW (2008). Contemporary outcomes after the Fontan procedure: A pediatric heart network multicenter study. J. Am. Coll. Cardiol..

[12] Ishizaki U (2019). Global strain and dyssynchrony of the single ventricle predict adverse cardiac events after the Fontan procedure: Analysis using feature-tracking cine magnetic resonance imaging. J. Cardiol..

[13] Kanngiesser LM (2022). Serial assessment of right ventricular deformation in patients with hypoplastic left heart syndrome: A cardiovascular magnetic resonance feature tracking study. J. Am. Heart Assoc..

[14] Vonk-Noordegraaf A (2013). Right heart adaptation to pulmonary arterial hypertension: Physiology and pathobiology. J. Am. Coll. Cardiol..

[15] Sanz J, Sánchez-Quintana D, Bossone E, Bogaard HJ, Naeije R (2019). Anatomy, function, and dysfunction of the right ventricle: JACC state-of-the-art review. J. Am. Coll. Cardiol..

[16] Friedberg MK, Redington AN (2014). Right versus left ventricular failure. Circulation.

[17] Khoo NS (2011). Novel insights into RV adaptation and function in hypoplastic left heart syndrome between the first 2 Stages of surgical palliation. jACC Cardiovasc. Imaging.

[18] Schäfer M (2018). Effect of electrical dyssynchrony on left and right ventricular mechanics in children with pulmonary arterial hypertension. J. Hear. Lung Transplant..

[19] Tello K (2019). Cardiac magnetic resonance imaging-based right ventricular strain analysis for assessment of coupling and diastolic function in pulmonary hypertension. JACC Cardiovasc. Imaging.

[20] Jing L (2016). Left and right ventricular dyssynchrony and strains from cardiovascular magnetic resonance feature tracking do not predict deterioration of ventricular function in patients with repaired tetralogy of Fallot. J. Cardiovasc. Magn. Reson..

[21] Frank B (2019). Novel measures of left ventricular electromechanical discoordination predict clinical outcomes in children with pulmonary arterial hypertension. Am. J. Physiol. Circ. Physiol..

[22] Wang CL (2010). Recoordination rather than resynchronization predicts reverse remodeling after cardiac resynchronization therapy. J. Am. Soc. Echocardiogr..

[23] Mondal T (2014). Prognostic implications of the systolic to diastolic duration ratio in children with idiopathic or familial dilated cardiomyopathy. Circ. Cardiovasc. Imaging.

[24] Alkon J (2010). Usefulness of the right ventricular systolic to diastolic duration ratio to predict functional capacity and survival in children with pulmonary arterial hypertension. Am. J. Cardiol..

[25] Friedberg MK, Silverman NH (2006). The systolic to diastolic duration ratio in children with heart failure secondary to restrictive cardiomyopathy. J. Am. Soc. Echocardiogr..

[26] Rumbinaite E (2016). Early and late diastolic strain rate versus global longitudinal strain at rest and during dobutamine stress for the assessment of significant coronary artery stenosis in patients with a moderate and high probability of coronary artery disease. Echocardiography.

[27] Hu L (2019). Assessment of global and regional strain left ventricular in patients with preserved ejection fraction after Fontan operation using a tissue tracking technique. Int. J. Cardiovasc. Imaging.

[28] Karlsen S (2019). Global longitudinal strain is a more reproducible measure of left ventricular function than ejection fraction regardless of echocardiographic training. Cardiovasc. Ultrasound.

[29] Meyer SL (2021). Sex differences in cardiac function and clinical outcome in patients with a Fontan circulation. Int. J. Cardiol. Congenit. Hear. Dis..

[30] Schuster A (2013). The intra-observer reproducibility of cardiovascular magnetic resonance myocardial feature tracking strain assessment is independent of field strength. Eur. J. Radiol..

