# Supplementary material for: Myocardial strain-curve deformation patterns after Fontan operation
Source: Sci Rep. 2023 Jul 24;13:11912. doi: 10.1038/s41598-023-39226-y (PMC10366156; doi:10.1038/s41598-023-39226-y)
Supplement: Supplementary file 1 — Supplementary Information. [file 41598_2023_39226_MOESM1_ESM.docx]

PCA of course requires equal-length feature vectors; here we consider each type of global strain (GCS or GLS) signal in a unique analysis. Strain-time curves $\varepsilon(t)$ are typically represented as discretized numerical 1-D vectors. To proceed with temporal normalization, the temporal differences for each individual subject $\Delta t_{i}$ (CMR derived temporal resolution) were adjusted to ${\Delta t}_{i}^{adj}= \Delta t_{i}/\bar{T}$ , where $\bar{T}$ represents the average heart cycle period within the given dataset. To obtain strain type and ventricle morphology specific (SLV or SRV) data matrices with equal length feature vectors, all strain-time curve signals were interpolated to 30 temporal phases prior to insertion into their specific data matrix. This step was required for only small proportion (N=5) of enrolled subjects as the number of acquired SSFP phases was set to 30. This also had the (intentional) consequence of normalizing their strain-time span to a unit length, with each actual temporal length being retained for later renormalization. While interpolation does not inherently increase the information content of a signal, previous experience suggests that this technique for aligning features across a temporal signal is effective.

These uniformly interpolated and time-normalized signal vectors $\boldsymbol{\varepsilon}$***_i_*** are then feature vectors (rows) in strain and ventricle morphology specific *m*-by-*n* matrices (patient number size by normalized strain-time vector length),

$\mathbb{M}_{m\times n}= \left[ \begin{matrix} - & \varepsilon_{1}^{T} & - \\ - & \varepsilon_{2}^{T} & - \\ - & \vdots& - \\ - & \varepsilon_{m}^{T} & - \end{matrix} \right]$ (1)

where each $\mathbb{M}$ served as the final matrix for principal component analysis (PCA) for investigation of that lead.

PCA yielded 30 principal component vectors **p**_i_, $i\in[1,$ 30], each with 30 coefficients or loadings, representing unique directions of variance in the strain-time curve signal. Also obtained were the proportion of the total variance that each vector explains and the score values *s_i_* for each patient that describe the proportion of each **p**_i_ present in the original strain signals as $\varepsilon_{i}=s_{i}\mathbf{p}_{i}^{T}$. The **p**_i_ vectors are sorted by the proportion of their variance (largest to smallest) and the largest vectors contributing towards 80-90% of the total variance are retained for further analysis. Then, to appreciate the effect of these principal component vectors on average strain-time curv $\bar{\boldsymbol{\varepsilon}}$ defined as the means of the columns of matrix $\mathbb{M}$, we generated vector-specific voltage shape variation curves $\boldsymbol{(\varepsilon}_{\mathbf{def}}^{\mathbf{k}}\boldsymbol{)}$ from individual **p**_i_ vectors as

$\boldsymbol{\varepsilon}_{\mathbf{def}}^{\mathbf{k}}\mathbf{=}\bar{\boldsymbol{\varepsilon}}\mathbf{+}k\bar{\boldsymbol{\varepsilon}}\mathbf{p}_{\mathbf{i}}$ (2)

where k represents a scaling factor k, $k\in\{0,1,2$} modulating the effect of the principal component vectors.
